# BOTH COGNITIVE IMPAIRMENT AND DEPRESSION MAY INCREASE THE RISK OF POOR MUSCLE FUNCTION IN OLDER PEOPLE

**DOI:** 10.1093/geroni/igad104.3204

**Published:** 2023-12-21

**Authors:** I-Ting Liu

**Affiliations:** E Da Hospital, Kaohsiung City, Kaohsiung, Taiwan (Republic of China)

## Abstract

Muscle loss might occur in aging, target organ failure, metabolic and endocrine disorders, etc. The aim of this study was to identify the high-risk group of low grip strength, a key component of sarcopenia. 100 subjects aged 65 or older who received Comprehensive Geriatric Assessment (CGA) and grip strength measurement were enrolled at EDA Hospital, Taiwan, form January 2022 to February, 2023 (IRB: EMRP-110-108). Exclusion criteria: unclear consciousness or unable to cooperate 、advanced disease. CGA and grip strength measurement were performed by well-trained nurses and geriatrician. Depressive mood was defined as Geriatric Depression Scale-5 ≧2 or with diagnosed depression. Cognitive impairment was defined as Short Portable Mental State Questionnaire (SPMSQ) < 8 (SPMSQ < 6 in those without education) or with diagnosed dementia. Multiple linear regression was constructed to assess the independent associated factors on increased average grip strength of bilateral hands. Univariate analysis showed male, weight, ADL significantly associated with greater grip strength. Multiple linear regression revealed CKD(ß= -7.70, 95%CI -13.35~-2.05, p=0.011), COPD(ß= -9.15, 95%CI -17.15~-1.17, p=0.027 ), cognitive impairment(ß= -7.67, 95%CI -12.66~-2.65, p=0.005) and depressive mood(ß= -8.39, 95%CI -13.46~-3.32, p=0.003) have detrimental effect on grip strength even after adjustment of age, sex, BMI, socioeconomics, chronic comorbidities, and other geriatric syndromes. Our results resemble another sarcopenia study in which poor muscle function, but not reduced lean muscle mass, was associated with late-life cognitive impairment. Therefore, early screening, education, and intervention of sarcopenia should be applied on high-risk population such as those with CKD, COPD, cognitive impairment, and depressive mood.

